# Factors associated with core competencies of diabetes management staff in community health service institutions: a cross-sectional analysis

**DOI:** 10.3389/fpubh.2026.1816665

**Published:** 2026-07-02

**Authors:** Yiming Xiang, Wei Xie, Zhou Liu, Ya Xiu Reng

**Affiliations:** 1 Minda Hospital of Hubei Minzu University, Enshi, China; 2 Hubei Provincial Key Laboratory of Occurrence and Intervention of Rheumatic Diseases, Enshi, Hubei, China; 3 Hubei Provincial Clinical Medical Research Center for Nephropathy, Enshi, Hubei, China; 4 The First Affiliated Hospital of Guizhou University of Traditional Chinese Medicine, Guiyang, China; 5 General Practice Department, Enshi Tujia and Miao Autonomous Prefecture Central Hospital, Enshi, Hubei, China

**Keywords:** community health service institutions, core competency, influencing factors, self-directed learning, self-efficacy

## 1 Introduction

Diabetes Mellitus (DM) is a major global public health challenge. According to the International Diabetes Federation (IDF) (1), an estimated 589 million adults aged 20–79 years worldwide were living with diabetes in 2024. China has the highest number, reaching 148 million, accounting for one-quarter of the global diabetic population (1). In 2021, diabetes directly caused 6.7 million deaths (12.2% of global deaths) (1). Diabetes management is therefore a significant public health and socioeconomic concern in China. Primary care plays a pivotal role: achieving glycemic control through primary care can reduce the relative risk of diabetes-related complications by 12% and microvascular complications by 25% (2).

Despite continuous refinements in diabetes management guidelines, primary healthcare services face substantial challenges. In economically underdeveloped regions, resource disparities, insufficient staff knowledge and skills, and lack of motivation for continuous learning further constrain management quality (3, 4). Notably, over one-third of China's community health service institutions currently have inadequate chronic disease management capacity (5). Existing research focuses mainly on tertiary hospitals or specialized models, leaving a gap in assessing core competencies of primary institutions.

This study aims to clarify the current status and determinants of core competencies among diabetes management personnel in community health service institutions in Western China, providing data-driven support for health administrative departments to formulate strategies for improving primary diabetes management capacity.

## 2 Methods

### 2.1 Study design

**Figure 1:**
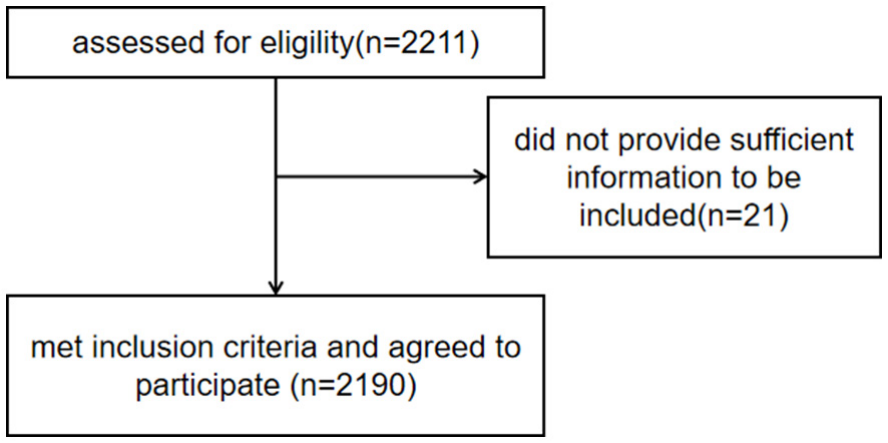
Flow chart of data screening.

This is a quantitative observational cross-sectional study (Figure 1).

### 2.2 Population and ethical approval

Inclusion criteria: (1) currently employed medical professionals in Guiyang community health institutions; (2) holding a valid practitioner certification; (3) ≥1 year of experience in diabetes management; (4) provision of informed consent. Exclusion criteria: (1) personnel engaged in long-term rotational training (≥6 months); (2) visiting staff; (3) declining participation. The study was approved by the Ethical Committee of The First Affiliated Hospital of Guizhou University of Traditional Chinese Medicine (Approval No. 138/2022). All participants provided written informed consent. The sample size was estimated as at least 638 based on 58 variables (6).

### 2.3 Procedures

A total of 48 community health institutions in Guiyang were enrolled. Data were collected via electronic survey links through WeChat. All completed questionnaires were double-checked; those with logical inconsistencies or >10% missing data were excluded. A total of 2,190 valid questionnaires were retained. The General Information Questionnaire included demographic and professional characteristics. The “Diabetes Specialist Nurse Core Competency Evaluation Indicator System Scale” (7) (48 items, Likert five-level) was used, with higher scores indicating stronger core competencies. The General Self-Efficacy Scale (GSE) (8) (10 items, four-point Likert) was used, with total scores ranging from 10 to 40.

### 2.4 Statistical analysis

Core competency scores were described with means and standard deviations, along with 95% confidence intervals (CI) for regression coefficients. Chi-square tests and one-way Analysis of Variance (ANOVA) were used to compare core competency scores across categorical variables. Multiple linear regression analysis was performed with total core competency score as the dependent variable. All analyses used SPSS 26.0 (IBM Corporation, Armonk, New York, USA).

## 3 Results

A total of 2,190 participants were included. Table 1 presents the comparison of core competency scores across demographic and professional characteristics. Significant differences were observed for education level (*F* = 17.691, *P* < 0.001), professional title (*F* = 4.207, *P* = 0.006), economic income (*F* = 7.012, *P* < 0.001), unit level (*F* = 53.788, *P* < 0.001), type of license (*F* = 27.042, *P* < 0.001), self-study or not (*t* = 10.379, *P* < 0.001), age (*F* = 29.207, *P* < 0.001), and years working with chronic diseases (*F* = 40.182, *P* < 0.001). No significant differences were found for sex (*P* = 0.624) or employment status (*P* = 0.187).

**Table 1:** Comparison of core competency scores among diabetes management personnel in community health service institutions by demographic and professional characteristics (*n* = 2,190).

Item	Frequency (*n*)	Percentage (%)	Core competency score (mean ±SD)	Statistical value (*F*/*t*)	*p*-value
Academic degree					
Secondary school	878	40.1	220.57 ± 44.17	17.691 (*F*)	< 0.001
College	887	40.5	208.05 ± 42.98		
Undergraduate	419	19.1	205.03 ± 40.77		
Graduate	6	0.3	203.17 ± 61.07		
Professional title					
Elementary	1,919	87.6	211.44 ± 43.71	4.207 (*F*)	0.006
Intermediate	244	11.1	218.26 ± 42.73		
Senior	27	1.3	233.12 ± 33.60		
Economic income (CNY/month)					
Below 3,000	1,248	57.0	214.12 ± 44.99	7.012 (*F*)	< 0.001
3,000–5,000	835	38.1	209.00 ± 41.64		
5,000–8,000	85	3.9	213.65 ± 38.93		
Above 8,000	22	1.0	246.91 ± 32.54		
Unit level					
Community health service center	529	24.2	196.55 ± 40.74	53.788 (*F*)	< 0.001
Community health service station	136	6.2	199.99 ± 35.51		
Health center	275	12.6	205.37 ± 41.54		
Village health office	1,250	57.1	222.14 ± 43.42		
Type of license					
Doctor	550	25.1	214.36 ± 42.72	27.042 (*F*)	< 0.001
Nurse	661	30.2	198.77 ± 40.89		
Physician assistant	886	40.5	221.32 ± 43.55		
Public health	64	2.9	212.97 ± 46.39		
Pharmacist	29	1.3	218.03 ± 39.93		
Self-study or not					
Yes	2,054	93.8	214.91 ± 42.70	10.379 (*t*)	< 0.001
No	136	6.2	175.79 ± 40.41		
Age (years)					
20 and below	7	0.3	204.57 ± 37.43	29.207 (*F*)	< 0.001
21–29	476	21.7	197.28 ± 40.38		
30–39	557	25.4	207.57 ± 43.68		
40–49	743	33.9	221.22 ± 43.06		
Above 50	407	18.6	221.14 ± 43.59		
Years working with chronic diseases					
1–3 years	811	37.0	202.40 ± 41.89	40.182 (*F*)	< 0.001
3–5 years	268	12.2	208.33 ± 44.00		
5–10 years	613	28.0	214.61 ± 41.98		
More than 10 years	498	22.7	228.49 ± 43.59		

^*^Statistical methods used: one-way ANOVA (F) for comparisons across ≥3 groups; independent t-test (t) for comparisons between two groups. ^*^Statistically significant difference at *p* < 0.05.

Table 2 shows that the overall core competency attainment rate (88.5%) significantly exceeded the general self-efficacy rate (74.3%). Health management ability (2.87 ± 0.69) and research ability (3.08 ± 1.22) were the lowest-scoring dimensions.

**Table 2:** Core competencies and general self-efficacy scores of diabetes managers in community health service organizations (*n* = 2,190).

Item	Total score (mean ±SD)	Entry mean score (mean ±SD)
Self-efficacy score (total)	29.72 ± 7.08	2.97 ± 0.71
Core competency (total)	212.48 ± 43.59	4.43 ± 0.91
Specialized nursing	50.09 ± 10.72	3.85 ± 0.82
Health education ability	61.87 ± 13.61	4.12 ± 0.91
Health management ability	11.47 ± 2.77	2.87 ± 0.69
Counseling ability	18.81 ± 4.59	3.76 ± 0.92
Coordination and communication ability	11.69 ± 2.60	3.90 ± 0.87
Research ability	12.33 ± 4.87	3.08 ± 1.22
Personal quality	16.49 ± 3.40	4.12 ± 0.85

Entry mean score = total score of the dimension divided by number of items in that dimension.

Table 3 presents the multiple linear regression analysis. Self-directed learning (*B* = −13.67, 95% CI: −18.65 to −8.69, β = −0.076, *P* < 0.001), years of working with chronic illness (*B* = 1.88, 95% CI: 0.60–3.16, β = 0.052, *P* = 0.004), and total self-efficacy score (*B* = 4.44, 95% CI: 4.27–4.61, β = 0.722, *P* < 0.001) were significant predictors. The adjusted *R*^2^ of the model was 0.528.

**Table 3:** Results of multiple linear regression analysis of factors influencing core competency of diabetes management personnel in community health service institutions (*n* = 2,190).

Independent variable	*B* (unstandardized)	SE	95% CI for *B*	Beta (standardized)	*t*	*P*-value
(Constant)	85.27	5.90	73.70–96.84		14.46	< 0.001
Age	0.44	0.80	−1.12 to 2.00	0.01	0.55	0.580
Years working with chronic illness	1.88	0.65	0.60–3.16	0.05	2.88	0.004
Total self-efficacy score	4.44	0.09	4.27–4.61	0.72	50.33	< 0.001
Education	−1.71	1.04	−3.75 to 0.33	−0.03	−1.65	0.099
Title	2.92	1.71	−0.44 to 6.28	0.03	1.70	0.089
Economic income	1.57	1.04	−0.46 to 3.60	0.02	1.52	0.129
Name of organization (unit level)	1.41	0.64	0.15–2.67	0.04	2.19	0.028
Previous job (employment status)	−1.42	0.74	−2.86 to 0.02	−0.03	−1.93	0.054
Self-study or not (reference: yes)	−13.67	2.54	−18.65 to −8.69	−0.08	−5.39	< 0.001

^*^Dependent variable = total core competency score. Adjusted R^2^ = 0.528. CI, confidence interval. Bold indicates statistical significance (*P* < 0.05). ^*^Statistically significant difference at *p* < 0.05.

## 4 Discussion

This study found that the total core competency score among diabetes management personnel was 212.48 ± 43.59, which exceeds results reported by Jinping et al. (9). Among all dimensions, health education ability and personal quality scores were higher, consistent with the extensive health education experience of community staff. However, health management ability scored the lowest (2.87 ± 0.69). This finding has important policy implications. In Guiyang and similar western regions, diabetes managers often handle multiple chronic diseases simultaneously due to uneven resource distribution and shortage of primary medical resources. Traditional management methods prevail, creating a gap with advanced scientific management. From a policy perspective, this suggests that training programs should move beyond basic education to include modern chronic care management models (e.g., Chronic Care Model, patient-centered medical homes). Second, research ability also scored low (3.08 ± 1.22), reflecting limited exposure to evidence-based practice. Community health administrators should establish research mentorship programs and simple data collection systems to encourage inquiry into local diabetes patterns.

Self-directed learning was positively associated with core competencies, similar to Qingyun et al. (10). Managers who engaged in self-directed learning had significantly higher scores (*B* = −13.67). This underscores the need for institutional policies that allocate protected time for self-learning and peer supervision mechanisms.

Longer working years were positively correlated with higher core competency scores, aligning with Xiaoli et al. (11) but differing from Ye et al. (12). In China, professional title promotion is linked to working years, and higher-title staff demonstrate better competencies (13). Thus, targeted training for junior staff is essential.

General self-efficacy showed the strongest association with core competencies (β = 0.722). Self-efficacy refers to belief and confidence in performing tasks and is a decisive factor for maintaining self-management behaviors such as continuous learning (14). This finding aligns with studies in other healthcare settings (15, 16). Managers should provide counseling support (e.g., “heart-to-heart rooms”) to improve self-efficacy.

Comparison with other settings: Compared to Shenzhen, a first-tier city with greater resources, Guiyang staff showed lower health management ability but comparable health education skills. This reflects the resource gradient within China. Internationally, studies from low- and middle-income countries (LMICs) such as India and Kenya report similar challenges—community health workers often lack structured competency frameworks and opportunities for continuing education. Our findings suggest that tailored, low-cost interventions (e.g., mobile-based micro-learning, peer coaching) could benefit both western China and other LMIC settings.

Policy implications: Based on these findings, we recommend: (1) integrating self-directed learning time into annual performance assessments; (2) developing tiered training modules focusing on health management and research competencies; (3) implementing self-efficacy enhancement programs including regular feedback and stress management; (4) establishing regional partnerships between tertiary hospitals and community stations to support knowledge transfer.

### 4.1 Limitations

This study has limitations. Convenience sampling limits generalizability. Self-reported data may introduce bias. The cross-sectional design precludes causal inference. The competency scale was developed for diabetes specialist nurses but our sample included physicians; its validity has not been formally established for non-nurse professionals.

## 5 Conclusions

Diabetes management staff in Guiyang's community health facilities possess moderate-to-high core competencies. Factors associated with these competencies included self-directed learning, years of experience in chronic disease management, and general self-efficacy. Community managers should encourage self-directed learning and design interventions to improve self-efficacy. Given the cross-sectional design, future longitudinal studies are required to test directional relationships.

## Data Availability

The original contributions presented in the study are included in the article/supplementary material, further inquiries can be directed to the corresponding authors.
